# Inscribing diffraction grating inside silicon substrate using a subnanosecond laser in one photon absorption wavelength

**DOI:** 10.1038/s41598-020-78564-z

**Published:** 2020-12-08

**Authors:** Kozo Sugimoto, Shigeki Matsuo, Yoshiki Naoi

**Affiliations:** 1 Department of Mechanical Engineering, Shibaura Institute of Technology, Toyosu, Koto-ku, Tokyo, 135-8548 Japan; 2 Graduate School of Technology, Industrial and Social Science, Tokushima University, Tokushima, 770-8506 Japan; 3 Institute of Post-LED Photonics, Tokushima University, Tokushima, 770-8506 Japan

**Keywords:** Laser material processing, Micro-optics

## Abstract

Using focused subnanosecond laser pulses at $1.064\,\upmu \hbox {m}$ wavelength, modification of silicon into opaque state was induced. While silicon exhibits one-photon absorption at this wavelength, the modification was induced inside $300\,\upmu \hbox {m}$-thick silicon substrate without damaging top or bottom surfaces. The depth range of the focus position was investigated where inside of the substrate can be modified without damaging the surfaces. Using this technique, diffraction gratings were inscribed inside silicon substrate. Diffraction from the gratings were observed, and the diffraction angle well agreed with the theoretical value. These results demonstrate that this technique could be used for fabricating infrared optical elements in silicon.

## Introduction

In recent years, ultrafast laser processing inside transparent solid materials has been attracting interest as a tool of three-dimensional (3D) micro-nano processing technique^1–3^. For example, marking, inscribing waveguide, and selective etching inside glass have been reported^4–7^. In these research, non-linear optical phenomena, such as multi-photon absorption, is utilized for localized modification of materials. Now application fields of ultrafast laser processing inside glass is expanding such as microphotonics and microfluidics^1–3^.

Silicon is one of the most important material in modern technology. Its application field includes large-scale integrated circuits (LSIs), micro-electro-mechanical systems (MEMS) devices, and multi-pixel photodetectors. In these, semiconductor technology based on photolithography, which is a two-dimensional technique, is mainly used for fabrication. Recently research on the use of silicon as a near-infrared (NIR) photonic platform material is active. For such applications, 3D processing using lasers will be advantageous.

Silicon is transparent in the near-infrared range (the band gap of silicon is 1.12 eV, and corresponding wavelength is 1.11 $\upmu \hbox {m}$), thus use of an ultrafast laser in this wavelength range seems promising. However, unlike glasses, difficulty has been reported to process inside silicon with an ultrafast laser in the transparent wavelength region^8^. Thus, special methods have been executed, such as use of optical setup with extremely high numerical aperture of 2.97^9^, double pulse^10^, and use of high repetition rate laser^11–13^. In the study by Matthäus et al.^12^, waveguides were inscribed in longitudinal geometry starting at the exit surface then moved upstream, in this case the waveguide inscription was significantly facilitated by an imperfectly flat exit surface^13^. Very recently, it was reported that temporal contrast (pre/post-pulse, pedestal), which is laser technology dependent, of ultrafast laser pulses has a significant effect on the modification threshold energy^14^. Investigation using THz-repetition-rate pulse bursts also showed the effectiveness of peak-suppressed consecutive pulses for laser modification inside silicon^15^.

In contrast to ultrafast lasers, longer pulse lasers are effective for modification inside silicon. Verburg et al. reported modification inside silicon using laser pulse of 1.549 $\upmu \hbox {m}$ wavelength and 3.5 ns duration^16^. Tokel et al. used reflection on the back surface and fabricated modification inside silicon with 1.550 $\upmu \hbox {m}$ and 5 ns pulses^17^. In their method the length of modification along the optical axis was controlled by the number of pulses. They also reported fabrication of waveguide, and selective etching of modified region. Kammer et al. made modifications inside silicon with 1.552 $\upmu \hbox {m}$ wavelength and duration ranging from 800 fs to 10 ps, and reported that 10 ps pulses showed better reproducibility^18^. Chambonneau et al. fabricated waveguides^19^ and gratings^20^ using 1.55 $\upmu \hbox {m}$ wavelength and 5 ns duration pulses, and estimated the degree of refractive index change^19,20^. Wang et al. inscribed waveguides that have symmetric cross-section in transverse geometry using 1.55 $\upmu \hbox {m}$ wavelength and 3.5 ns duration pulses^21^. Investigation on pulse-duration dependence was carried out by Das et al., and they reported that pulse duration of 5.4 ps or more is required for making modifications at NA = 0.85^22^.

Generally, one-photon absorption is not used for laser processing inside solid materials. Meanwhile, in cutting silicon substrate using internal modification, a Nd:YAG laser operating at 1.064 $\upmu \hbox {m}$ wavelength is used^23^, at this wavelength there is weak but non-negligible one-photon absorption (absorption coefficient of silicon at 1.064 $\upmu \hbox {m}$ about 9.7 cm$^{-1}$ at 295 K)^24^. However, there have been no report on the use of one-photon absorption based fabrication for optical applications. One-photon absorption based fabrication could be more efficient than that based on multi-photon absorption, whereas there are possible drawbacks that energy loss due to absorption in pre-focal region, and limitation in the inscribing depth due to damaging surface and/or pre-focal region. In addition, a Nd:YAG laser operating at 1.064 $\upmu \hbox {m}$ is widely used. Thus it is worth investigating to apply one-photon absorption based fabrication in modification inside silicon. In this research, we used a subnanosecond Nd:YAG laser operating at 1.064 $\upmu \hbox {m}$ wavelength, and inscribed diffraction gratings inside silicon substrate without damaging top or bottom surfaces.

## Results and discussion

### Inscribing inside silicon substrate without damaging surfaces

**Figure 1:**
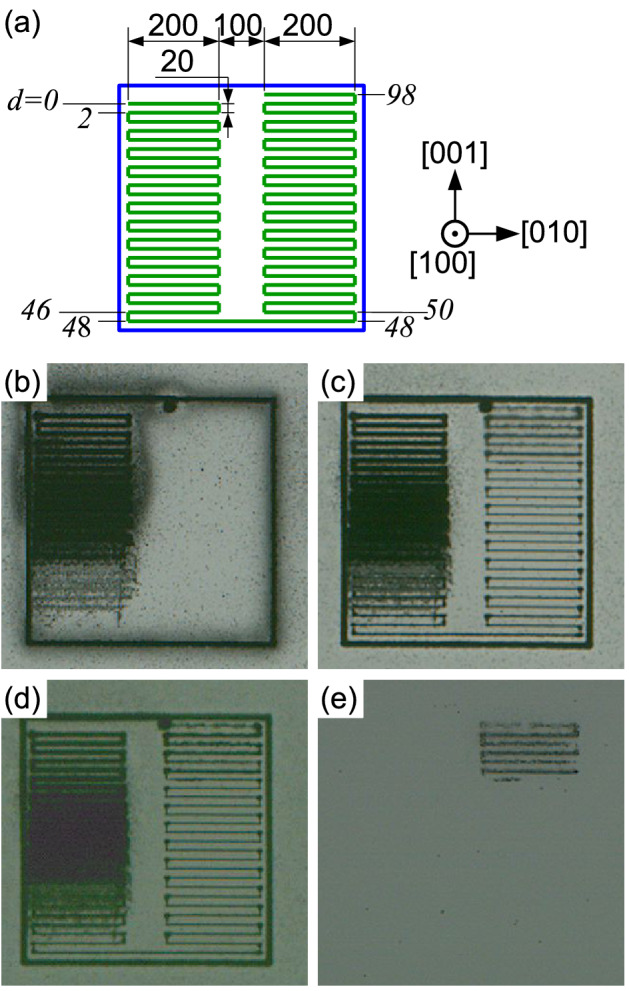
Results of laser-inscribing inside silicon substrate as a function of the position of focus in the sample. (**a**) scheme of inscribing; all the numbers are in micrometre. The italic numbers shows ascending distance *d* (see text). Blue rectangle indicates the lines inscribed on the top surface. (**b**) top view, reflected illumination. (**c**) Top view, transmitted illumination. (**d**) Backside view, transmitted illumination. (**e**) Backside view, reflected illumination. Here, ‘backside view’ indicates that the substrate was inverted and observed from above, and the images were flipped so that the geometry agrees with that of the top views.

**Figure 2:**
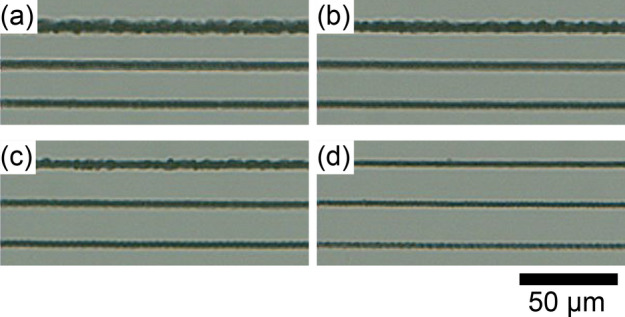
Pulse energy and scanning speed dependence of modified lines inscribed at $d=60$ $\upmu \hbox {m}$. Pulse energy was (**a**) 8.8 $\upmu \hbox {J}$, (**b**) 6.6 $\upmu \hbox {J}$, (**c**) 4.4 $\upmu \hbox {J}$, and (**d**) 2.2 $\upmu \hbox {J}$. In each panel, scanning speed was 30, 50, and 70 $\upmu \hbox {m}/\hbox {s}$ from top to bottom.

At first, we examined the condition where we can inscribe modified lines inside silicon substrate without damaging top or bottom surfaces. For this, lines parallel to the surface were inscribed at different depths, experimentally sample position along optical axis was changed for each line, as shown in Fig. 1(a). Hereafter the sample position along optical axis is referred to as ascending distance *d*; $d=0$ indicates the focus of laser was set at the top surface of silicon substrate, and positive value of *d* indicates the sample was moved to the laser source (accordingly, the focus was moved to the bottom surface by roughly $d \times n_{Si}$; $n_{Si}$ is the refractive index of silicon). After inscribing the lines with an objective lens of numerical aperture (NA) = 0.65, the substrate was observed by the NIR microscope under transmitted or reflected illumination. Results with pulse energy of 8.8 $\upmu \hbox {J}$ and scanning speed of 50 $\upmu \hbox {m}$/s are shown in Fig. 1(b)–(e). The inscribed lines appeared dark. As seen in the images with transmitted illumination (Fig. 1(c) and (d)), modification (including surface damage) was induced in whole range of *d*. Surface damages are seen in the images with reflected illumination. As seen in Fig. 1(b) top surface was damaged in $d<45$ $\upmu \hbox {m}$, and bottom surface was damaged in $d>85$ $\upmu \hbox {m}$ as seen in Fig. 1(e). Accordingly, only inside the substrate was modified without damaging top or bottom surfaces in the range about $d=45$–85 $\upmu \hbox {m}$. Similar results were obtained with the other pulse energies and scanning speeds. Figure 2 shows pulse energy and scanning speed dependence of modified lines at $d=60$ $\upmu \hbox {m}$, where the lines were inscribed without damaging surfaces with all parameters shown here. The thickness of the lines was in the range of 2–6 $\upmu \hbox {m}$ depending on the pulse energy and scanning speed. The higher pulse energy or the slower scanning speed, the thicker the lines. In addition, some of the lines had irregularity in width.

**Figure 3:**
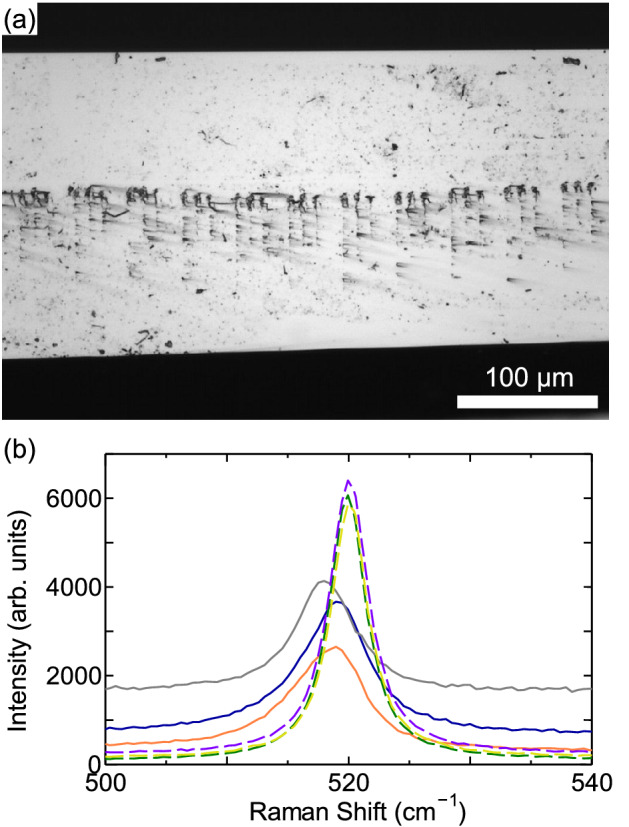
(**a**) Optical micrograph of cleaved face of silicon substrate. The substrate was cleaved perpendicular to the laser-inscribed lines. (**b**) Micro-Raman spectra of modified and un-modified spots around 520 cm$^{-1}$. Three solid curves are spectra from modified spots, and three dashed curves are spectra from un-modified spots.

### Characterization of modification inside silicon substrate

Cross-section of silicon substrate with inscribed lines was observed. The irradiation condition was the same as that in Fig. 1 (8.8 $\upmu \hbox {J}$, 50 $\upmu \hbox {m}$/s), and *d* was set at 52 $\upmu \hbox {m}$. The substrate was cleaved perpendicular to the inscribed lines, As seen in Fig. 3(a), a series of modified spots aligned in vertical direction (that is, the direction of laser irradiation) is observed. The presence of the vertically aligned spots indicates that completely 3D-localized modification was not realized yet, but the modification was localized in two-dimensions thus can be used in two-dimensional applications. Such a series of modified spots have been reported in nanosecond laser processing of silicon^25,26^. In these studies, modified spots were identified as voids. Li et al. explained that this phenomena is due to void formation and hydrodynamic phenomena occurring near the void induced by successive laser pulses^25^. We presume that similar phenomenon occurred in our case, while laser wavelength was different. Another possibility might be a phenomena related to the propagation of the absorption front to the upstream of the incoming laser^27,28^. In this case, however, the speed of propagation is about 1 $\upmu \hbox {m}$ $/ 1$ ns or less, thus it is difficult to explain the total length of the observed modified spots considering the pulse duration of 0.5 ns in the present study.

In order to elucidate the nature of modified spots, micro-Raman spectra were measured at modified and un-modified spots (three spots each) on the cleaved face. Figure 3(b) shows the spectra around 520 cm$^{-1}$. Compared to the spectra from un-modified spots, the spectra from modified spots are broader and shifted to lower Raman shift. These changes can be explained by polycrystallization^29,30^ or strain^31,32^.

### Grating and diffraction

**Figure 4:**
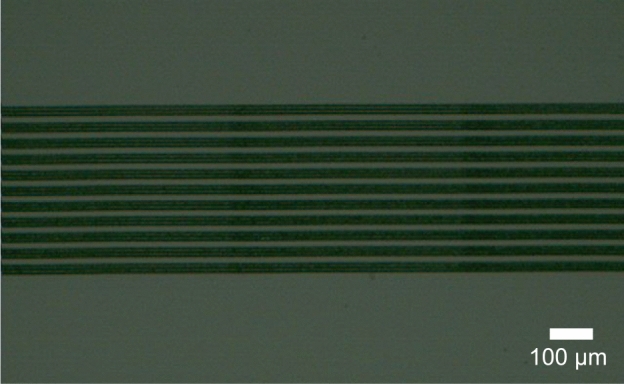
Grating inscribed inside silicon substrate (top view, transmitted illumination).

Several gratings with different period and width of lines were inscribed. Pulse energy was 8.8 $\upmu \hbox {J}$, scanning speed was 50 $\upmu \hbox {m}$/s, and *d* was 60 $\upmu \hbox {m}$ for all. Figure 4 shows one of the gratings observed with the NIR microscope transmitted illumination. Here 11 dark lines were inscribed with a lattice constant of 34 $\upmu \hbox {m}$. The width of the dark lines were about 24 $\upmu \hbox {m}$; each line consists of six paths of inscription with a separation of 4 $\upmu \hbox {m}$. As seen in Fig. 4, grating was inscribed inside silicon substrate as designed. In addition, this transmitted illumination image indicates that the inscribed grating is, at least in part, an amplitude grating, whereas Chambonneau et al. evaluated only the real part of the refractive index change in the laser-modified zone^20^.

**Figure 5:**
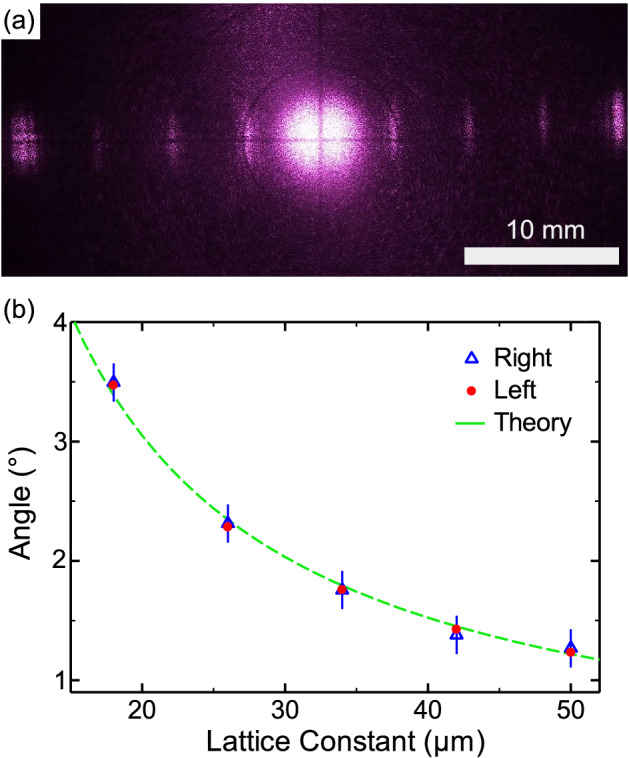
(**a**) Diffraction pattern from the grating in Fig. 4. The distance between the grating and the target paper was 150 mm. The dark lines in the image are the marks written on the target paper (see Methods). (**b**) Dependence of first-order diffraction angle on the lattice constant. The width of the dark lines was varied, so that the width of the transparent lines was kept constant at 10 $\upmu \hbox {m}$.

Diffraction pattern from the gratings were observed in transmission geometry. Figure 5(a) shows the diffraction pattern from the grating of Fig. 4. The distance between the grating and the target paper was set at $L=150$ mm. As seen, in addition to the strong zero-order line, diffraction lines up to 4-th order were observed. In this case, theoretically, first-order diffraction should be found at $\pm 4.7$ mm ($=L \tan \theta$, where $\theta =\sin ^{-1} ( \lambda / \Lambda )$ is the first-order diffraction angle, $\lambda$ is the laser wavelength, and $\Lambda$ is the lattice constant). The experimental result is in good agreement with the theoretical one. Dependence of first-order diffraction angle on the lattice constant is shown in Fig. 5(b). As seen, diffraction angle from the other gratings also agreed with the theory well. These results indicate that the fabricated gratings inside silicon substrate functioned as expected.

## Conclusion

We have inscribed diffraction gratings inside 300 $\upmu \hbox {m}$-thick silicon substrate using a subnanosecond laser of 1.064 $\upmu \hbox {m}$ wavelength. Although silicon exhibits non-negligible one-photon absorption at this wavelength, the gratings were inscribed without damaging top or bottom surfaces. The functionality of the inscribed gratings were demonstrated. So far, internal modification inside silicon substrate by laser pulse of 1.064 $\upmu \hbox {m}$ wavelength has been used for dicing. The present results indicate that the modification based on one-photon absorption can also be used for optical applications. Silicon is one of important infrared optical materials. This technique would be useful for fabricating amplitude optical elements in silicon.

## Methods

N-type Si (100) substrates with a thickness of 0.3 mm, mirror-polished on both sides, were cut into 10 mm$\times$8 mm pieces, and used for experiments.

The light source used for laser-inscribing was a subnanosecond Nd:YAG laser (PNP-M08010-130, Teem Photonics) with a wavelength of 1.064 $\upmu \hbox {m}$ and a pulse duration of 0.5 ns. There are no pre-pulses or pedestals, and there could be post-pulses with a contrast of 1:10 (post-pulse:main-pulse energy). The repetition rate was 100 Hz for all the experiments shown in this article. The laser beam was led to a near-infrared (NIR) upright optical microscope (BX-51, Olympus), and focused by an objective lens with correction collar (LCPLN50XIR, Olympus, numerical aperture (NA) = 0.65) from the top. The theoretical spot size is ${1.22 \lambda }/{NA}=2.0$ $\upmu \hbox {m}$. The correction collar settings was fixed to 0.3; this value was chosen because empirically it gives a reasonable focus on all range of depth. The laser pulse energy was measured at a fixed point in front of the microscope and multiplied by the overall transmission. The sample was placed on a three-axis motorized stage (MMU-60X$\cdot$Y and ALZ-6012-G0M, Chuo Precision Industrial) and moved along the pre-programmed pattern.

**Figure 6:**
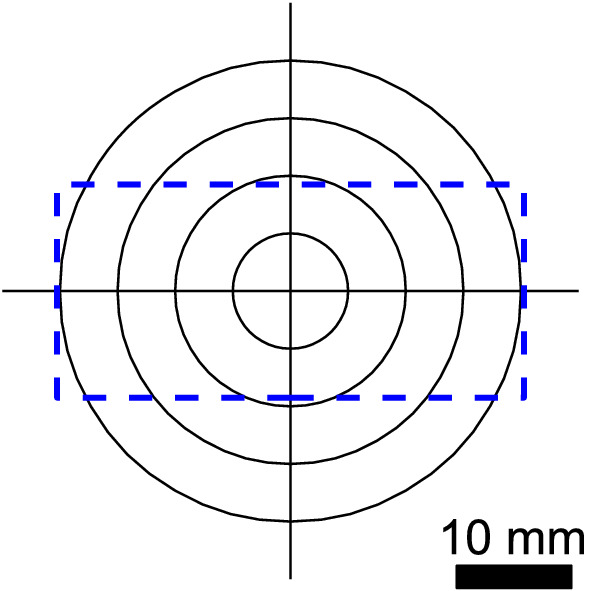
The mark printed on the target paper. The blue-dashed rectangle approximately indicates the region shown in Fig. 5.

To observe the laser inscribed lines inside the substrate, a NIR camera (CONTOUR-IR digital, Electrooptic) was attached on the same microscope, and observed under transmitted or reflected illumination. Under transmitted illumination the dark (opaque) region in whole thickness of the substrate was observed, while under reflected illumination only the top surface was observed. In both transmitted/reflected illumination, the light source was a halogen lamp (U-LH100IR, Olympus).

A confocal Raman microscope (LabRAM HR Evolution, Horiba) was used for measuring Micro-Raman spectra.

For the observation of diffraction pattern from the fabricated gratings, non-focused laser beam at 1.064 $\upmu \hbox {m}$ wavelength was irradiated to the grating at normal incidence, and the pattern of transmitted (diffracted) light on a target paper was recorded by a digital camera (E-PM2, Olympus). The camera has week sensitivity at 1.064 $\upmu \hbox {m}$; laser beam appeared violet in the recorded images. Because of the observation angle, the image was slightly distorted. Thus, a cross mark and concentric circles were printed on the target paper as show in Fig. 6.
